# Depressive symptoms in first episode psychosis: a one-year follow-up study

**DOI:** 10.1186/1471-244X-13-106

**Published:** 2013-04-05

**Authors:** Nasrettin Sönmez, Kristin Lie Romm, Ole A Andreasssen, Ingrid Melle, Jan Ivar Røssberg

**Affiliations:** 1 Oslo University Hospital, (TIPS Sor Ost) Centre of Competence for Early Intervention in Psychosis, Fridtjof Nansens vei 12, Oslo, 0369, Norway; 2 KG Jebsen Centre for Psychosis Research, Division of Mental Health and Addiction and Oslo University Hospital, Institute of Clinical Medicine, University of Oslo, Pb 85, Oslo, 0319, Norway

## Abstract

**Background:**

Depressive symptoms are common in patients with first episode psychosis (FEP) and have serious consequences for them. The main aims of this study were to examine the course of depression in FEP patients and explore whether any patient characteristics at baseline predicts depressive symptoms after one year.

**Method:**

A total of 198 FEP patients with schizophrenia spectrum disorders were assessed for depressive symptoms with Calgary Depression Scale for Schizophrenia (CDSS) at baseline and 127 were followed for one year. A CDSS score [greater than or equal to] 6 was used as a cut-off score for depression.

**Results:**

Approximately 50% of the patients were depressed (CDSS[greater than or equal to]6) at baseline. At follow-up approximately 35% had depression. The course of depressive symptoms varied, 26% was depressed at both baseline and follow-up, 9% became depressed during the follow-up, 22% remitted from depression during the 12 months and 43% was neither depressed at baseline nor at follow-up. Poor childhood social functioning, long duration of untreated psychosis (DUP) and depressive symptoms at baseline predicted depression at 12 months follow-up.

**Conclusion:**

Depressive symptoms are frequent in the first year after onset of psychosis. Patients with poor social functioning in childhood, long DUP and depressive symptoms at baseline are more prone to have depressive symptoms after one year. These patients should be identified and proper treatment provided.

## Background

Depressive symptoms are common in patients with a first episode psychosis (FEP) with a reported prevalence from 17% to 83% in the different studies [1-4]. This wide variation in prevalence may be due to heterogeneity in the study population, variation of assessment tools and the study’s definition of depression. Depressive symptoms occur in different phases of the psychosis, including prodromal [5,6], acute [7] and post-psychotic phases [8,9]. They have thus been regarded as intrinsic to schizophrenia psychopathology, similar to positive, negative, and disorganized symptom clusters [10].

There are only few studies examining the course and development of depressive symptoms in patients with a FEP. This is important as there is an increased risk of suicide during the early phases of psychotic illness [11,12]. Furthermore, knowledge about the course of depressive symptoms may facilitate earlier identification of patients who are prone to develop a severe course. In line with this, Cotton and coworkers [13] emphasized the need for prospective studies mapping the trajectory of depressive symptoms in psychotic disorders to provide a critical step towards developing a clear understanding of the phenomenology of depressive pathology in the early phases of psychotic disorders.

To our knowledge only five studies have explored the course of depression in FEP over time; and two of these were acute treatment outcome studies which only explored the course of depressive symptoms from admission to discharge (between 39 and 56 days) [3,14]. Riedel and colleagues [14] found that depressed first-episode schizophrenia patients scored significantly higher on all PANSS subscales (PANSS total, PANSS positive and PANSS negative) both at admission and discharge. However, Bottlender and coworkers [3] found that depressive symptoms in the acute psychotic phase was a positive prognostic indicator for patients concerning negative symptoms.

Oosthuizen and coworkers [15] assessed changes in depressive symptoms over a two-years period. They found that depressive symptoms experienced during an acute psychotic episode were different from those experienced in the post-psychotic period. Acute depressive symptoms were related to psychotic symptoms, and these symptoms improved when the psychotic symptoms resolved in response to antipsychotic treatment. Depressive symptoms observed in the post-psychotic period were referred to as ‘persistent depressive symptoms’, because they were not responsive to antipsychotic therapy alone.

Cotton and colleagues [13] aimed to determine if there were differences in the clinical and functional characteristics of FEP patients with and without depressive symptoms during an18 months early intervention treatment program [16]. The group with depressive symptoms at baseline was less hospitalized and had less substance abuse during treatment. At discharge patients in this group were also more likely to be depressed and had better insight concerning their illness. The depressed group of patients was also significantly more likely to have a past diagnosis of personality disorder, suicide attempts, a shorter duration of treatment, ongoing substance abuse, lower GAF mean score and were less likely to be working.

Upthegrove and colleagues [17] examined prospectively the course of depression in prodromal, acute, and 12 months follow-up period of psychosis. Including a total of 82 patients, they found that prodromal depression was significantly linked with the development of depression in the acute and follow-up phases, and acute depression was also significantly related with the development of later depression. Severity of depression was not significantly correlated with the severity of positive and negative symptoms in the acute or follow-up phases.

As seen from this review there is a need for a wider and more elaborated examination of the association between the course of depressive symptoms, patient characteristics and outcome.

In the present study we examined the course of depressive symptoms during a 12 month follow-up period.

We aimed to answer the following questions:

1. How many FEP patients are scored as depressed measured by CDSS (CDSS ≥ 6) at baseline and at 12 months follow-up?

2. Is it possible to identify subgroups of patients with different profiles of depressive symptoms during a one-year follow-up period, and can these subgroups be differentiated on demographic characteristics such as DUP, premorbid functioning (PAS) and clinical characteristics (PANSS scores)?

3. To what extent are there any differences in suicidality, between these groups?

4. To what extent do patient characteristics at baseline predict 12 months CDSS scores?

## Method

### Subjects

A total of 198 patients with a first episode psychosis (FEP) were included at baseline from the ongoing Thematically Organized Psychosis Research (TOP) study at the Oslo University Hospital and University of Oslo, Norway. Of these, a total of 127 patients were followed for one year. Romm and colleagues have previously reported the baseline characteristics of 119 of the 198 patients included in the present study [4]. The present study is a further exploration of depressive symptoms in a 12 months follow-up period.

These samples were included from March 2004 to February 2010. The inclusion criteria were (1) age 18 to 65 years and a first episode non-affective psychosis according to Diagnostic and Structural Manual of Mental Disorders, fourth edition (DSM-IV) [18]. Exclusion criteria were a history of organic brain disorder, a significant comorbid medical condition, or an IQ below 70.

Inclusion was based on the time from the first onset of positive psychotic symptoms (the first week with a PANSS score of 4 or more on Positive Scale Items 1, 3, 5, 6 or General Scale item 9) to the start of first admission to the study [19]. Patients with previous treatment with antipsychotic medication in adequate dosage for more than 12 weeks were not considered as FEP patients.

The study is approved by the Regional Committee for Medical Research Ethics and the Norwegian Data Inspectorate. A written informed consent was obtained from all of the participants.

### Clinical assessment and instruments

Diagnosis was set by the Structural Clinical Interview for DSM-IV axis I disorders (SCID I) [18]. Current symptom severity was assessed on the Positive and Negative Syndrome Scale (PANSS) [20] and characterized by five subscales; positive, negative, excitative, depressive, and cognitive [21]. Depression was assessed using the Calgary Depression Scale for Schizophrenia (CDSS) [22]. CDSS has been shown to be reliable and valid and it measures depression separate from negative or extrapyramidal symptoms [23,24]. In a systematic review of instruments measuring depressive symptoms, the CDSS outperformed other depression instruments in terms of reliability and validity in patients with schizophrenia [25]. The cut-off score for depression varies between three and nine in the different studies [15,17,26-28]. We chose to use a cut-off score of equal and/or above six, in line with some of the previous studies [28-30]. Suicidality was measured with the CDSS-suicide item. Premorbid functioning was assessed with the Premorbid Adjustment Scale (PAS) [31]. The premorbid phase is defined as the time from birth until six months before onset of psychosis. PAS measures social and academic functioning in 4 time periods; childhood, early adolescence, late adolescence, and adult life. We decided to focus on social and academic functioning in childhood and early adolescence only. It may be difficult to discern between premorbid and psychotic symptoms in late adolescence when the first psychotic episode usually occur. General level of symptoms and functioning was assessed with the Global Assessment of Functioning scale (GAF), split version [32,33]. Problem drinking was screened by the Alcohol Use Disorders Identification Test (AUDIT) [34], and use of illegal drugs was assessed with Drug Use Disorders Identification Test (DUDIT) [35]. DUP was measured following the criteria described by Larsen et al. [36]. At 12-month follow-up the participants were re-assessed with the following instruments: GAF, PANSS, CDSS, AUDIT and DUDIT.

### Reliability

Clinical interviews were carried out by trained psychologists and psychiatrists. The interviewers were all enrolled in the general training and reliability program in the TOP study. For DSM-IV diagnostics, mean overall kappa with training videos was 0.77 (95% confidence interval [CI]: 0.60-0.94). Interrater reliability, measured by the interclass correlation coefficient (1.1), was for the PANSS positive subscale 0.82 (95% CI: 0.66-0.94), for the PANSS negative subscale 0.76 (95% CI: 0.58-093), the PANSS general subscale 0.73 (95% CI: 0.54-0.90), the GAF symptom scale 0.86 (95% CI: 0.77-0.92), and the GAF functioning scale 0.85 (95% CI: 0.76-0.92).

### Analysis

Statistical analysis was done by using SPSS for Windows (version 16.0). Descriptive statistics was performed on the whole sample to obtain the independent variables’ frequencies, means and standard deviations. To identify different subgroups of patients with different trajectories of depressive symptoms, we used a cut-off score above or equal six on the CDSS. Based on the CDSS scores we wanted to identify patients who were depressed in the whole follow-up period, patients who were depressed at baseline, but recovered during the follow-up period, patients who became depressed during the follow-up period, and finally patients who never were scored as depressed during the follow-up period. One-Way ANOVA was performed in order to compare the means of the follow-up subgroups. Bivariate correlations were used to study the correlation of each independent variable with CDSS total scores at both baseline and 12-month follow-up.

DUP required transformation to its natural logarithm (Ln[DUP + 1]).

Hierarchical multiple linear regression was used to assess the ability of baseline variables to predict depression (CDSS total score) at 12-month follow-up. The predictors were selected based on theoretical considerations: gender differences, neurocognitive developmental issues (premorbid functioning in childhood), and basic psychopathological/psychotic symptoms that may have association with the development of depressive symptoms in FEP. To estimate how much of the variance each of the patient variables explained, we used a blockwise multiple hierarchic regression analysis. Gender was entered in the first block, because this variable must be considered as the most basic one. PAS childhood social and academic were entered in the second block as a measure of early social and academic functioning. The duration of psychosis was entered in the third block. Finally, we entered the five different PANSS subscales which we considered as the most important variables in predicting depressive symptoms at 12 months follow-up. By entering the PANSS subscale scores in the fourth block, we controlled for the amount of variance explained by the variables in the three first blocks.

## Results

Table 1 displays the baseline characteristics of the patients. The mean age at baseline was 27.98 years, the range was from 17 to 56, and 127 (64%) of the sample were men. A total of 152 (77%) patients were European (at least one parent), 34 were married/cohabited (17%), 118 (77%) had high school and 74 (37%) were working or studying. A total of 60 (30%) patients lived alone and 57 (29%) were living with their parents. The diagnostic distribution was as follows: schizophrenia 102 ( 51.5%), schizophreniform disorder 17 ( 8.5%), schizoaffective disorder 10 ( 5.0%), brief psychotic disorder 8 ( 4.0%), delusional disorder 9 ( 4.5%), psychotic disorder not otherwise specified 52 ( 26.5%).

**Table 1:** Mean and SD of patients’ demographic and clinical baseline characteristics and their correlations with CDSS total scores at baseline (n = 198) and 12-month follow-up (n = 127)

	**Mean (SD)**	**Correlation With CDSS total score at baseline (Pearson)**	**Correlation with CDSS total score at12-month follow-up (Pearson)**
Age	27.98 (8.70)	0.01	0.10
Gender (M)	N = 127 (64%)	0.18*	0.13
Education (year)	12.71 (2.93)	−0.02	−0.13
GAF symptom	41.12 (11.38)	−0.29**	−0.26**
GAF function	44.14 (12.65)	−0.11	−0.22*
PANSS positive	12.95 (4.49)	0.07	0.06
PANSS negative	20.13 (7.72)	0.11	0.18*
PANSS excitative	8.07 (2.84)	0.21*	−0.04
PANSS depressive	12.15 (3.61)	0.58**	0.37**
PANSS cognitive	5.38 (2.07)	−0.004	0.06
PANSS-total score	63.38 (15.00)	0.27**	0.23*
PAS childhood social	2.51 (3.05)	0.26**	0.32**
PAS childhood academic	3.45 (2.43)	0.26**	0.26**
PAS early adolescence social	2.69 (2.88)	0.21**	0.22*
PAS early adolesc. academic	4.26 (2.75)	0.24*	0.17
DUPln	1.63 (0.80)	0.23**	0.30**
Insight (PANSS G12)	2.76 (1.36)	−0.18*	−0.11
AUDIT	7.76 (7.70)	0.17*	0.09
DUDIT	6.36 (9.58)	0.10	0.03
Anti-depressive medication day dosis	37.50 (35.75)	0.05	0.03
Anti-psychotic medication, day dosis	97.17 (155.88)	−0.05	−0.07
CDSS total baseline	6.23 (4.44)	--------------------	0.48**

*Correlation is significant at the 0.05 level (2-tailed).

**Correlation is significant at the 0.01 level (2-tailed).

*PANSS* Positive and negative syndrome scale.

*GAF* Global assessment of functioning.

*PAS* Premorbid adjustment scale.

*DUP* Duration of untreated psychosis.

*AUDIT* Alcohol use disorders identification test.

*DUDIT* Drug use disorders identification test.

*CDSS* Calgary depression scale for schizophrenia.

A total of 96 (48.5%) patients had a score of CDSS ≥ 6 at baseline. At follow-up 44 (34.6%) patients had a score above or equal six. The associations between these patients’ demographic and clinical characteristics and their CDSS total score at baseline and at 12 months follow-up are also presented in Table 1.

CDSS at baseline correlated significantly with GAF S, PANSS excitative, PAS childhood social and academic, PAS early adolescence social and academic, DUP, Insight (PANSS g12) and AUDIT. Among the demographic variables there was only gender that correlated significantly with CDSS-total at baseline (female most depressed). At 12 months follow-up CDSS correlated significantly with baseline GAF S and GAF F, PANSS negative and depressive component, PAS childhood social and academic, and PAS early adolescence social, and DUP.

The course of depressive symptoms during the 12 months period followed four different pathways: Group 1) thirty-three patients (26%) were depressed at both baseline and follow-up (G1; Persistent depression), Group 2) eleven patients (9%) had no depressive symptoms at baseline, but developed depressive symptoms during the 12-months follow-up (G2; Depression follow-up), Group 3) twenty-eight patients (22%) had depressive symptoms at baseline and remitted during the 12 months follow-up (G3; Depression baseline), Group 4) fifty five patients (43%) had no depressive symptoms neither at baseline nor at follow-up (G4; No depression) (Table 2).

**Table 2:** Comparison of the 4 subgroups regarding baseline variables

	**G1: Persistent depression**	**G2: depression follow-up**	**G3: depression baseline**	**G4: No depression**	**Significant difference(s) between;**
	**N = 33**	**N = 11**	**N = 28**	**N = 55**	
Gender (M)	18/55%	8/73%	15/54%	41/75%	
	Mean (SD)	Mean (SD)	Mean (SD)	Mean (SD)	
Age	28.3 (8.71)	28.0 (5.90)	23.9 (5.00)	27.2 (7.81)	
GAF symptom	37.58 (8.58)	39.36 (8.61)	38.93 (8.58)	45.31 (14.87)	G1 and G4 (.034)
GAF function	40.24 (12.37)	43.00 (11.81)	42.39 (11.93)	47.53 (14.34)	
PANSS Positive	13.52 (4.24)	13.45 (5.26)	13.61 (4.68)	12.76 (4.89)	
PANSS Negative	21.83 (8.06)	21.36 (7.75)	21.93 (7.89)	18.78 (6.84)	
PANSS Excitative	7.88 (1.90)	7.00 (2.10)	8.66 (2.94)	7.55 (2.41)	
PANSS Depressive	14.79 (2.10)	13.55 (4.53)	12.96 (2.59)	9.89 (3.09)	G1 and G4 (.000)
					G2 and G4 (.003)
					G3 and G4 (.000)
PANSS Cognitive	5.67 (2.16)	5.36 (2.16)	5.39 (1.93)	5.38 (1.87)	
PANSS-total	68.50 (12.22)	65.91 (11.90)	67.78 (14.90)	58.56 (13.34)	G1 and G4 (.013)
					G3 and G4 (.038)
AUDIT	8.87 (8.63)	4.70 (7.45)	9.21 (5.63)	6.46 (7.53)	
DUDIT	7.16 (11.18)	2.40 (4.60)	5.21 (8.59)	7.27 (10.03)	
PAS Childhood social	4.09 (3.75)	2.36 (2.54)	1.96 (2.60)	1.69 (2.24)	G1 and G4 (.003)
					G1 and G3 (.038)
PAS Childhood academic	4.29 (2.44)	4.45 (3.21)	3.54 (2.76)	2.55 (1.92)	G1 and G4 (.017)
PAS Early adolescence social	4.06 (3.55)	2.27 (2.57)	2.71 (2.68)	2.05 (2.44)	G1 and G4 (.020)
PAS Early adolesc. academic	4.71 (2.83)	4.82 (3.06)	4.25 (3.04)	3.22 (2.30)	
DUPin	4.17 (1.76)	3.86 (1.78)	3.44 (1.85)	3.35 (1.68)	
CDSS-suicide	.91 (.678)	.18 (.405)	.75 (.645)	.11 (.315)	G1 and G4 (.000)
					G1 and G2 (.002)
					G2 and G3 (.035)

*PANSS* Positive and negative syndrome scale.

*GAF Global Assessment of Functioning*.

*PAS* Premorbid adjustment scale.

*DUP* Duration of untreated psychosis.

*AUDIT* Alcohol use disorders identification test.

*DUDIT* Drug use disorders identification test.

*CDSS* Calgary depression scale for schizophrenia.

Table 2 shows the differences between the four groups at baseline. As displayed, the Persistent depression group (G1) scored significantly different compared to the group no depression. (G4) concerning GAF S, PAS childhood social, academic and PAS early adolescence social. Regarding CDSS suicide score at baseline, G1 had significantly higher score than both G2 and G4, and G3 had significantly higher suicidality score than G2.

Table 3 displays the differences between the four groups at 12 months follow-up. The most notable difference was, between G1 and G4. G1 had higher levels of global symptoms (GAF S), poorer functioning (GAF F) and more negative and excitative symptoms (PANSS components). Regarding CDSS suicide score, G1 had significantly higher score at follow-up than all the other three subgroups.

**Table 3:** Comparison of the 4 subgroups regarding 12 months follow up variables

	**G1: Persistent depression**	**G2: Depression follow-up**	**G3: Depression baseline**	**G4: No depression**	**Significant difference(s) between;**
	**N = 33**	**N = 11**	**N = 28**	**N = 55**	
Gender (M)	18/54%	8/73%	15/54%	41/75%	
	Mean (SD)	Mean (SD)	Mean (SD)	Mean (SD)	
Age	28.33 (8.71)	28.00 (5.90)	23.86 (5.00)	27.20 (7.81)	
GAF symptom	40.21 (10.45)	40.45 (10.37)	58.52 (15.66)	54.36 (17.10)	G1 and G4 (.001)
					G1 and G3 (.000)
					G2 and G4 (.049)
					G2 and G3 (.011)
GAF function	42.42 (11.22)	43.82 (13.35)	57.48 (15.58)	56.60 (16.07)	G1 and G4 (.000)
					G1 and G3 (.002)
PANSS Positive	12.65 (4.74)	13.64 (6.03)	9.24 (3.97)	10.42 (5.57)	
PANSS Negative	21.24 (7.12)	19.73 (7.39)	16.71 (6.31)	16.57 (5.54)	G1 and G4 (.013)
PANSS Excitative	7.76 (2.56)	7.18 (2.44)	6.54 (1.67)	6.46 (2.02)	G1 and G4 (.064)
PANSS Depressive	13.70 (3.11)	13.64 (3.38)	8.29 (2.21)	8.74 (3.15)	G1 and G4 (.000)
					G1 and G3 (.000)
					G2 and G4 (.000)
					G2 and G3 (.000)
PANSS Cognitive	5.61 (2.51)	5.82 (1.54)	4.93 (2.28)	4.93 (2.03)	
PANSS-total	65.82 (13.29)	64.36 (12.21)	49.11 (12.08)	50.67 (13.76)	G1 and G4 (.000)
					G1 and G3 (.000)
					G2 and G4 (.023)
					G2 and G3 (.017)
AUDIT	9.29 (7.40)	3.40 (4.97)	4.88 (4.65)	6.35 (7.31)	
DUDIT	5.55 (8.04)	.20 (.63)	4.04 (5.60)	4.58 (8.65)	
CDSS-suicide	.79 (.740)	.36 (.505)	.04 (.189)	.02 (.135)	G1 and G4 (.000)
					G1 and G2 (.045)
					G1 and G3 (.000)

*PANSS* Positive and negative syndrome scale.

*GAF* Global assessment of functioning.

*PAS* Premorbid adjustment scale.

*DUP* Duration of untreated psychosis.

*AUDIT* Alcohol use disorders identification test.

*DUDIT* Drug use disorders identification test.

*CDSS* Calgary depression scale for schizophrenia.

A blockwise multiple regression analysis was conducted in order to detect possible variables at baseline that might predict depressive symptoms at 12 months follow-up. The included variables explained a total of 25% of the variance in the level of depression as measured by the CDSS. PAS childhood social, DUP, and PANSS depressive symptoms explained a significant proportion of the variance (Table 4).

**Table 4:** Blockwise multiple regression analysis of the relationship between baseline characteristics and CDSS total score

	**B**	**Standard error**	**Significance**	**Lower bound**	**Upper bound**	**Adjusted R square**
Gender	.703	.660	.289	-.603	.289	.009
PAS child. social	.319	.118	.008*	.085	.552	
PAS child. academic	.044	.142	.755	-.237	.325	.111
DUP	.558	.181	.003*	.198	.917	.171
PANSS positive	-.057	.071	.420	-.198	.083	
PANSS negative	.039	.46	.397	-.053	.132	
PANSS excitative	-.248	.130	.060	-.506	.010	
PANSS depressive	.348	.093	.000**	.165	.532	
PANSS cognitive	.046	.177	.797	-.305	.396	.251

Explained variance for final model: R^2^ =.305, F=5.608, P<.05.

*Significant at the P<.05 level.

**Significant at the P<.001 level.

*PANSS* Positive and negative syndrome scale.

*PAS* Premorbid adjustment scale.

*DUP* Duration of untreated psychosis.

*CDSS* Calgary depression scale for schizophrenia.

## Discussion

The present study showed that depressive symptoms are frequent during the first year of treatment in FEP. Approximately half of the patients had depression at start of treatment while more than one third were depressed at one-year follow-up. In line with other studies [8,13,14,17] the depressive symptoms decreased during the follow-up period.

Furthermore, we detected four different trajectories of depressive symptoms during a 12 months follow-up. The four groups differed significantly as it comes to patient characteristics and clinical variables. The most notably difference was between G1 (persistent depression) and G4 (no depression). G1 scored significantly higher on global symptoms in addition to premorbid adjustments (PAS childhood social and academic score, and PAS early adolescence social score) at baseline. At 12 months follow-up the differences between the two groups were even more pronounced. Most notably the group with persistent depression scored poorer on GAF F, and the PANSS negative symptom scale.

As it comes to suicidality the group with persistent depression (G1) was, as expected, more suicidal than the other groups. However, there were also significant differences in level of suicidal thoughts at follow-up between this group (G1) and the group with depression follow-up (G2), in spite of the fact that they had equal levels of depression. As shown in table three the PANSS depressive component was almost the same between the two groups.

Birchwood and colleagues [9] emphasized the need for making clear distinctions between three core but not mutually exclusive pathways to depression in schizophrenia; a) depression that is intrinsic to the psychosis diathesis, b) depression as a reaction to the psychotic episode and c) depression as a product of disturbed developmental pathways resulting from developmental trauma and the childhood antecedents of psychosis. Based on the current findings, one could speculate that the group with *persistent depression* (G1) corresponds to the pathway that explain depressive symptoms in psychosis as intrinsic to the psychosis diathesis [9]. This type of depression is seen as an essential aspect of the schizophrenic process being an artefact of negative symptoms and Parkinsonism in a limited subgroup of patients whom these symptoms are particularly severe [37]. Accordingly, G1 had the highest PANSS-negative score and significantly higher score than the G4’s PANSS-negative score.

Upthegrove and colleagues emphasizes how some patients with a psychotic disorder may be more vulnerable to persistent depression. According to them, there may be a depression that begins in adolescence, presents itself during the prodromal phase and re-emerges at future times as a response to varied stressors [17]. This is to some extent in line with the current findings in G1. This group seems to be more vulnerable not only to depression, but shows more global and overall psychological symptoms and has the lowest premorbid function (childhood social, academic and early adolescent social). Vulnerability to depression for this group may be partly due to the severity of these symptoms. That this group did not significantly differ from G2 and G3 in positive and negative symptoms may indicate that depression in FEP is not only *intrinsic* to psychotic symptoms, but also a reaction to the more acute constraint of general and global psychological symptoms which develop during the psychotic episode.

To our knowledge, the present study is the first to examine if baseline socio-demographic and clinical characteristics predict depressive symptoms 12 months after start of therapy. Patients with poor premorbid social functioning in childhood, long duration of untreated psychosis and depressive symptoms at baseline have more depressive symptoms at 12 months follow-up. The group with persistent depression (G1) had most global and psychotic symptoms, as well as the highest suicide scores at both baseline and follow-up. It is of great importance to recognize such a group of FEP patients as early as possible, in order to provide a better prognosis and to prevent depressive symptoms and suicidality. Riedel and colleagues [14] examined predictors of depression in the acute phases of psychosis in patients treated with risperidone and haloperidol. However, they did not, as in the present study, examine patient characteristics that could predict depressive symptoms after the acute phases of the psychoses. Upthegrove and co-authors [17] found that depressive symptoms in the acute phase of psychosis predicted depressive symptoms at 12 months follow-up. This is in line with the findings in our study. In addition, we show that a long DUP and poor premorbid social functioning in childhood predicts depressive symptoms at 12 months follow-up. Premorbid functioning has previously been associated with long term treatment outcome [38-42] However, this is the first study that have showed that premorbid functioning is important for the development of depressive symptoms in FEP patients.

### Limitations

#### Limitation of the study concern drop-out

The results in the present study could be influenced by the relatively high drop-out rate (n = 71, 36%) from baseline to 12 months follow-up. This represents loss of valuable information and may weaken the validity of the findings. The patients who dropped out were most often untraceable and did not respond to letters or phone calls. Unfortunately, no additional information about reasons for dropping out from the study was registered. However, there were no significant differences at baseline between those patients who dropped-out and the group who were interviewed at 12 months follow-up as it comes to gender distribution, age, symptoms and diagnostic distribution.

It could be argued that the results of the present study are influenced by the overlap between negative symptoms and depressive symptoms. However, the present study revealed no significant correlation between the PANSS negative component scores and the CDSS total scores at baseline and only a low but significant correlation (0.18) between these two measures at 12 months follow up. Even if we can not rule out an overlap between these negative and depressive symptoms, it would probably have no major consequences for the results.

This study has not measured any qualitative aspects by being depressed. The group of patients who were continously depressed could have different reasons for being depressed at respectively baseline and 12 months follow up. Factors like not being able to attend school or work, might cause depressive symptoms just after the onset of psychotic symptoms. Furthermore, negative voices putting the patients down, internal and external stigma might cause depressive symptoms. These aspects and how they are related to the course of depressive symptoms in patients with psychosis should be explored in future studies. In the present study, we examined whether some of the items comprising CDSS were scored differently at baseline and 12 months follow up for the continously depressed patients. No significant differences were found.

## Conclusion

Depressive symptoms are frequent in FEP patients, both at baseline and 12 months follow-up. Patient characteristics such as premorbid social functioning, duration of untreated psychosis and PANSS score of depression at baseline predict depressive symptoms at 12 months follow-up. Patients with different depressive symptoms trajectories differed on both baseline socio-demographic and clinical characteristics. These findings may be useful for improving identification and management of depressive symptoms in FEP patients.

### Study design

1) Prevalence of depressive symptoms in two different measurement times; A baseline versus B 1-year.

2) Exploration of the course of depressive symptoms during the 1-year follow-up.

## Abbreviations

FEP: First episode psychosis; PANSS: Positive and negative syndrome scale; GAF F: Global assessment of functioning, function scale; GAF S: Global assessment of functioning, symptom scale; CDSS: Calgary depression scale for schizophrenia; DUP: Duration of untreated psychosis; PAS: Premorbid adjustment scale; TOP: Thematically organized psychosis research; DSM: Diagnostic and structural manual of mental disorders; AUDIT: Alcohol use disorders identification test; DUDIT: Drug use disorders identification test; ANOVA: Analysis of variance.

## Competing interest

This study was supported by *ExtraStiftelsen Helse og Rehabilitering*. The founding source had no further role in the study design, the collection, analysis and interpretation of data, the writing of the report or the decision to submit the paper for publication.

All authors declare that they have no conflict of interest.

## Authors’ contributions

NS: Data analysis, drafting and revising the manuscript; KLR: , Data analysis, conception of the study and revising the manuscript; OAA: conception of the study, collecting data, and revising the manuscript; IM: conception of the study and collecting data, revising the manuscript; JIR: conception of the study and collecting data, analysis, drafting and revising the manuscript. All authors read and approved the final manuscript.

## Pre-publication history

The pre-publication history for this paper can be accessed here:

http://www.biomedcentral.com/1471-244X/13/106/prepub
